# Performance of hybrid gain formula versus traditional fitting formulas in hearing aid fitting in tinnitus patients with hearing loss

**DOI:** 10.1007/s00405-024-08846-z

**Published:** 2024-07-28

**Authors:** Eser Sendesen, Hasan Colak

**Affiliations:** 1 Department of Audiology, Hacettepe University, Ankara, Turkey; 2 Biosciences institute, Newcastle University, Newcastle Upon Tyne, UK

**Keywords:** Tinnitus, Hearing aid, Hearing aid fitting, Speech perception

## Abstract

**Purpose:**

Hearing aid fitting can be challenging when tinnitus accompanies hearing loss, as speech intelligibility and quality of life are affected by both hearing loss and tinnitus perception. However, studies focusing on the optimal hearing aid fitting for this group are scarce. Here, we aim to investigate the performance of alternative hearing aid fitting scenarios in improving hearing aid benefit and managing tinnitus.

**Methods:**

Sixty-six participants were included in the study and randomly divided into three groups based on the fitting formula: NAL-NL2, DSL pediatric and hybrid gain fitting procedure (covering NAL-NL2 for low frequencies and DSL pediatric formulas for high frequencies). Hearing aid benefit was evaluated using the Abbreviated Profile of Hearing Aid Benefit (APHAB) questionnaire and speech perception in noise (SPIN). To evaluate tinnitus perception, psychoacoustic characteristics of tinnitus were determined, and the Tinnitus Handicap Inventory (THI) was gathered.

**Results:**

The NAL-NL2 fitting procedure showed better results in hearing aid benefit and SPIN compared to the DSL pediatric procedure. In the DSL pediatric procedure, better results were obtained in tinnitus management compared to NAL-NL2. There was no difference between the hybrid fitting procedure and DSL pediatric in tinnitus management. The hybrid fitting procedure also did not differ from NAL-NL2 in SPIN and hearing aid benefit.

**Conclusion:**

Here, we propose a hybrid gain fitting procedure that can be a better alternative to boost hearing aid performance and tinnitus management in clinical practice.

**Supplementary Information:**

The online version contains supplementary material available at 10.1007/s00405-024-08846-z.

## Introduction

Tinnitus is a phantom sound perception caused by central auditory system activity [1]. It has been estimated that 10–15% of the population is affected by tinnitus [2, 3]. A global study found that tinnitus affects more than 740 million people, and hearing loss is also present in about 80% of these cases [4]. It has been demonstrated that peripheral hearing loss causes changes in the central auditory system [5]. These changes likely predispose to the perception of tinnitus. One putative mechanism suggests that damaged cochlear hair cells and unbalanced input to the dorsal cochlear nucleus lead to abnormal spontaneous activity [6]. This abnormal spontaneous neural activity is perceived as tinnitus.

It is widely accepted that hearing aids (HAs) are an effective treatment for hearing loss. Previous studies recommend HAs as a tinnitus management strategy in hearing impaired patients [7–9]. However, HA benefit on tinnitus has been reported to vary between 30% and 80% across studies [10]. One reason for this variability may be differences in HA fitting.

Two methods are commonly used in HA fitting: National Acoustical Laboratories’ non-linear fitting procedure (NAL-NL) [11] and Desired Sensation Level (DSL) [12]. NAL-NL uses a loudness-normalization technique to optimize speech intelligibility and normalize the overall loudness. On the other hand, DSL was developed based on a loudness-equalization technique to equalize the loudness for each frequency channel separately. Comparing the two methods, it is shown that the DSL fitting procedure has higher gain than NAL-NL in each frequency response [13]. The effect of these two methods on tinnitus has also been investigated in studies. Wise [14] compared the effect of DSL (input/output (i/o)) v4 and NAL-NL1 on tinnitus and showed that DSL(i/o) was more effective than NAL-NL1 in tinnitus management. As a drawback, the author reported that patients were disturbed by environmental noise in the DSL(i/o) formula. Therefore, the author suggested using the DSL formula for suppressing tinnitus with environmental sounds and the NAL-NL formula to improve speech comprehension performance. Another study found that the DSL (i/o) v5 formula was more efficient than the NAL-NL2 formula in tinnitus management [15]. It has been noted that for both formulas, applying additional gain at the tinnitus frequency (less for DSL (i/o) v5 than for NAL-NL2) increases the success in tinnitus management.

The pediatric version of the DSL is a revised formulation of the DSL based on the needs of children who differ from adults in terms of auditory system maturation and auditory perception [12]. This formula was designed to provide more gain per frequency channel than DSL (i/o) v5 [16, 17]. Similar to Shetty and Pottackal [15], animal studies showed that sound enrichment in hearing impaired frequencies has a tinnitus suppressive effect by reducing hyperactivity in the auditory system [18, 19]. Therefore, it is plausible to argue that DSL pediatric fitting formula can be an effective management option in tinnitus patients. However, the increased gain of DSL formulas and excessive amplification of environmental sounds may negatively affect speech perception, especially in adverse listening conditions. Here, we propose a new hybrid gain formula that encompasses the NAL-NL2 formula for pre-1 kHz (frequency range where environmental sound frequencies are dominated) [20, 21] and the DSL pediatric formula for the following frequencies. Thus, it is hypothesized to provide improved speech perception and tinnitus management. The aim of this study was to evaluate the effects of NAL-NL2, DSL pediatric and the proposed hybrid gain formula on speech perception in noise (SPIN), tinnitus management and self-reported HA benefit.

## Materials and methods

### Participants

The study included sixty-six patients aged 18 to 50 with chronic tinnitus (> 6 months) and bilateral symmetrical sensorineural hearing loss. All participants were bilateral hearing aid users. Participants were recruited from the audiology clinic of the university hospital and, all study procedures were conducted in the same clinic. Participants were divided randomly to three groups according to their fitting formula (NAL-NL2, DSL pediatric, hybrid gain formula). Participants were blinded to their adjusted fitting procedure. The audiologist who performed the assessment of each group was different from the researcher who performed the HA fitting of the participants. In this way, this study was blinded to the audiologist who performed the assessment. Twenty-two participants (12 males and 10 females) were fitted with NAL-NL2, 21 (9 males and 12 females) with DSL pediatric and 23 (11 males and 12 females) with hybrid gain formula. All participants were using RIC HAs and were new HA users. The target gain of the hybrid gain fitting formula was determined according to the NAL-NL2 formula for frequencies lower than 1 kHz and according to the DSL pediatric formula for frequencies higher than 1 kHz. Since the noise components and environmental sounds are mostly below 1 kHz [22, 23], this frequency was chosen as the cut-off frequency in hybrid gain fitting formula.

Participants underwent otoscopy and tympanometry examinations and all participants had normal middle ear function. Patients with objective tinnitus, conductive hearing loss, and patients who have any psychiatric or neurologic history were excluded. Patients with severe (> 70 dB HL) or profound hearing loss (> 90 dB HL) according to the pure tone average or those who could not obtain a hearing threshold at any of the frequencies from 0.125 to 8 kHz were also excluded from the study. By doing so, patients for whom the HA was able to provide adequate gain at all frequencies in accordance with the fitting procedure were included in the study. Minimum masking level (MML), tinnitus handicap inventory (THI), SPIN and the abbreviated profile of HA benefit (APHAB) scores of the participants were evaluated at pre-fitting, first month post-fitting, and third month post-fitting.

### Tinnitus assessment

A calibrated Interacoustics AC-40 audiometer and TDH-39P headphones were used to determine psychoacoustic characteristics of tinnitus. Tinnitus frequency was elicited by using a two-alternative forced-choice procedure with stimuli presented at 30 dB sensation level (SL) between 0.125 kHz and 20 kHz to prevent confusion with the auditory stimulus presented with tinnitus. The MML was determined in 5 dB steps as the level at which tinnitus was masked by presenting narrowband noise at the tinnitus frequency [24]. Participants with bilateral or central tinnitus were presented with narrowband noise on both sides of their heads. In the case of unilateral tinnitus, it was presented to the tinnitus ear. MML was assessed when participants were without HAs.

The THI was gathered to assess the impact of tinnitus on the daily lives of the patients [25]. The THI consists of 25 questions, and it provides data about the patients’ subjective psychological effects of tinnitus. It can be used to assess functional, emotional, and disruptive subscales of tinnitus. The maximum score that can be obtained is 100 and higher scores indicate more tinnitus related handicap in daily life.

### Hearing aid programming and real ear measurement (REM)

Hearing aids were fitted using the NAL-NL2 and DSL pediatric fitting formulas with the hearing aid-specific module of the NOAH software (HIMSA, Copenhagen, Denmark). The hybrid gain formula was fitted by selecting NAL-NL 2 from the HA module. Thus, the NAL-NL 2 fitting formula was used as a reference for variables such as knee points and compression time, and the DSL pediatric formula was used as a basis for gains only at high frequencies (> 1 kHz). For all participants, noise reduction and feedback reduction were turned off, and omnidirectional microphone tuning was enabled.

The Interacoustics Affinity 2.0 (Interacoustics, Assems, Denmark) system was used to match the gain of the HA to the target gain real ear insertion response (REIR). The REIR was calculated by subtracting the real ear aided response (REAR) from the real ear unaided response (REUR) at frequencies between 0.125 kHz and 8 kHz. A twenty-second long International Speech Test Signal (ISTS) signal at 50 dB SPL (mild sound) was presented as the input signal for all measurements. Thus, it aims to provide sufficient sound enrichment by focusing on mild sounds. Using Affinity software, the gains of HAs that used the NAL-NL 2 and DSL pediatric formulas confirmed the target gains of each formula. Participants who adequately reached the target gains specified in REM at all frequencies were included in the study. The hybrid gain fitting formula’s (previously applied to the NAL-NL2 fitting formula from the HA module) target gain was determined using Affinity, based on the NAL-NL2 formula’s target gain curve for frequencies less than 1 kHz and the DSL pediatric formula’s target gain curve for frequencies greater than 1 kHz.

### Speech perception in noise (SPIN)

SPIN evaluation of the participants conducted with a word-in-noise test by using GSI-61 audiometer (Grason-Stadler, Eden Prairie, USA). A 25-word monosyllabic word list [26] was used as the speech stimulus. This word list consists of three different phonemically balanced subcomponents: List A, List B, and List C. We used List A to measure the pre-hearing aid condition. Lists B and C were used to measure the first- and third-month follow-ups, respectively, to rule out potential training-related effects on the SPIN performances. Speech stimuli were presented at 65 dB SPL and speech noise at 60 dB SPL. Thus, participants’ SPIN performance was evaluated at 5 dB SNR. Participants were seated one meter away from the loudspeaker at an azimuth angle of 0 degrees in a soundproof room. The number of words that the participants could repeat correctly was multiplied by four and their scores out of 100 were calculated.

### The abbreviated profile of hearing aid benefit (APHAB)

The APHAB is a self-report questionnaire in which participants rate the frequency of their difficulty in various conditions [27]. It consists of four subscales: ease of communication in a quiet environment (EC), background noise (BN), and reverberation situations (RV). Participants also rate how often they react negatively to environmental sounds (AV) or how frequently they avoid environmental sounds. Total scores are calculated by sum of the EC, BN, and RV subscale scores. Higher scores indicate increased satisfaction with the HA.

### Statistical methods

The G*Power program (Heinrich-Heine-Universität Düsseldorf, Düsseldorf, Germany) was used to determine the sample size to be included in the study. Based on the mean and standard deviation values from the pilot study groups, the effect size (H1 coefficient) for the variable requiring the highest sample size (MML) was determined to be 0.25. To detect a clinically significant difference with a 5% type I error level and a minimum power of 85%, this study should include 21 participants in each group. The SPSS version 26 (IBM Inc., Armonk, NY, USA) package program was used to evaluate the data. Statistical analyses were performed using SPSS version 26 software. The conformity of the variables to normal distribution was analysed visually (histograms and probability plots) and analytically (Kolmogorov-Smirnov/Shapiro-Wilk tests). Descriptive analyses were performed using means and standard deviations for normally distributed variables. Time-dependent changes of THI, MML, SPIN, APHAB scores within and between groups (NAL-NL 2, DSL pediatric and hybrid gain fitting groups were considered as between-subjects factors) were analyzed using Repeated Measures ANOVA. THI, MML, SPIN, APHAB scores parameters were compared between different fitting method groups (NAL-NL 2, DSL pediatric and hybrid gain fitting groups) using one-way ANOVA test. Pairwise post-hoc comparisons were performed using Tukey test. Independent samples t-test was used for age variables. Statistically significant results were considered when the p-value was less than 0.05.

## Results

### Descriptive statistics

**Fig. 1:**
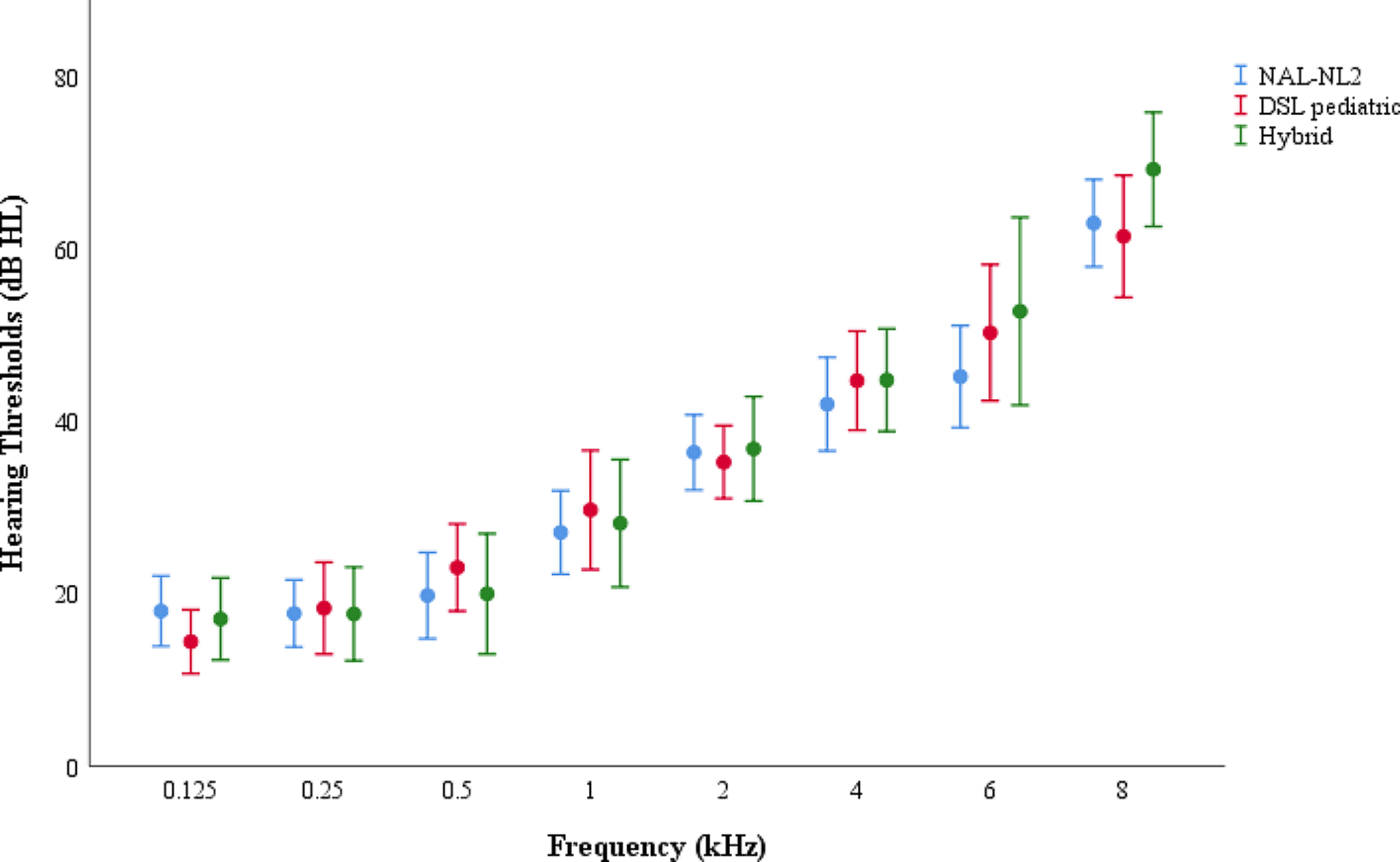
The mean hearing thresholds of the fitting groups

The mean ages of the NAL-NL 2, DSL pediatric and hybrid gain fitting groups were 37 ± 4.3, 34 ± 5.2 and 38 ± 3.9 years, respectively. Age differences between groups were not significant *(t(61) = 0.56*, *p* = 0.77). All pure-tone thresholds (PTTs) were not significantly different between groups (F(7,63) = 0.61, *p* = 0.29) for each frequency range (0.125–8 kHz). Tinnitus frequencies of participants ranged from 3 to 8 kHz, and there was no statistically significant difference between groups (F(2,63) = 0.187, *p* = 0.83). Tinnitus patients using the NAL-NL2 formula used 12 Phonak, 6 Resound, and 4 Oticon; those using the DSL pediatric formula used 13 Phonak, 5 Resound, and 3 Oticon; and those using the Hybrid formula used 15 Phonak, 6 Resound, and 2 Oticon HAs. The mean hearing thresholds of the fitting groups are shown in Fig. 1.

### Minimum masking level (MML)

We did not observe a statistical difference in Pre-HA MML values among all three fitting groups (F(2, 63) = 0.002, *p* = 0.99). The mean MML values were found to be 52.27 ± 12.79, 52.14 ± 15.69, and 52.17 ± 17.04 for NAL-NL2, DSL pediatric, and Hybrid fittings, respectively. We found significant reductions in MML from pre-HA to the first and third months across all fitting procedures (F(2, 63) = 3.19, *p* = 0.04, *r* = 0.09). While the MML values at the third month did not differ significantly for DSL pediatric (18.09 ± 7.82) and Hybrid (17.60 ± 8.51) fitting procedures (*p* = 0.98), the NAL-NL2 procedure (28.18 ± 9.09) showed significantly higher MML values than both DSL pediatric and Hybrid procedures (*p* < 0.01). Table 1 shows the change of MML over time and statistical comparison.

**Table 1:** Tinnitus perception over time for each fitting procedure

	Fitting Procedure			Multiple Comparisons (Tukey HSD)			
	NAL-NL2 (*n* = 22)	DSL Pediatric (*n* = 21)	Hybrid (*n* = 23)	NAL-NL2- DSL Pediatric	NAL-NL2- Hybrid		DSL Pediatric-Hybrid
MML (SPL)	Mean ± SD			*P* value			
**Pre-HA**	52.27 ± 12.79	52.14 ± 15.69	52.17 ± 17.04	*1.00*	*1.00*	*1.00*	
**First month**	42.04 ± 10.31	35.00 ± 11.51	34.13 ± 12.93	*0.12*	*0.67*	*0.96*	
**Third month**	28.18 ± 9.09	18.09 ± 7.82	17.60 ± 8.51	***< 0.01***	***< 0.01***	*0.98*	
**P value**	< 0.01*	< 0.01*	< 0.01*				
**THI**	**Mean ± SD**			**P value**			
**Pre-HA**	58.72 ± 13.30	57.71 ± 13.46	54.17 ± 18.24	*0.97*	*0.57*	*0.72*	
**First month**	28.54 ± 5.52	22.47 ± 3.62	23.86 ± 6.01	***< 0.01***	***0.01***	*0.65*	
**Third Month**	21.63 ± 6.12	10.66 ± 3.75	12.17 ± 3.80	***< 0.01***	***< 0.01***	*0.54*	
**P value**	< 0.01*	< 0.01*	< 0.01*				

n: number of participants, MML: Minimum Masking Level, THI: Tinnitus Handicap Inventory, * repeated measures

### Tinnitus handicap inventory (THI)

We observed no statistically significant differences in Pre-HA THI scores among the three fitting groups (F(2, 63) = 0.55, *p* = 0.57). The mean THI scores were found as 58.72 ± 13.30, 57.71 ± 13.46, and 54.17 ± 18.24 for the NAL-NL2, DSL pediatric, and Hybrid fitting groups, respectively. Significant reductions in THI scores were obtained from pre-HA to both the first and third months across all fitting procedures (F(2, 63) = 16, *p* < 0.001, *r* = 0.33), indicating a subjective decrease in tinnitus-related handicap. While the THI scores at the third month did not show significant differences for the DSL pediatric (10.66 ± 3.75) and Hybrid (12.17 ± 3.80) fitting procedures (*p* = 0.54), the NAL-NL2 fitted group (21.63 ± 6.12) displayed significantly higher THI scores compared to both the DSL pediatric and Hybrid procedures (*p* < 0.01). Table 1 shows the change of THI scores over time and statistical comparison.

### Speech perception in noise (SPIN)

The mean SPIN scores were not statistically significant in the Pre-HA condition for all three fitting groups (F(2, 63) = 0.25, *p* = 0.78), with mean SPIN performances found as 60.18 ± 6.23, 59.23 ± 7.54, and 60.69 ± 6.89 for the NAL-NL2, DSL pediatric, and Hybrid fitting groups, respectively. Significant reductions in SPIN scores were obtained from pre-HA to both the first and third months across all fitting procedures (F(2, 63) = 7.86, *p* = 0.01, *r* = 0.2). There was no significant difference at the third month between the NAL-NL2 (77.90 ± 6.30) and Hybrid (77.82 ± 4.96) procedures in terms of SPIN performance (*p* = 0.99). However, the DSL pediatric group (68.76 ± 7.91) exhibited significantly lower performance than both the NAL-NL2 and Hybrid groups (*p* < 0.01). Table 2 shows the change of SPIN test scores over time and statistical comparison.

**Table 2:** SPIN and hearing aid benefit over time for each fitting procedure

	Fitting Procedure			Multiple Comparisons (Tukey HSD)			
	NAL-NL2 (*n* = 22)	DSL Pediatric (*n* = 21)	Hybrid (*n* = 23)	NAL-NL2- DSL Pediatric	NAL-NL2- Hybrid		DSL Pediatric-Hybrid
SPIN (%)	Mean ± SD			*P* value			
**Pre-HA**	60.18 ± 6.23	59.23 ± 7.54	60.69 ± 6.89	*0.89*	*0.96*	*0.76*	
**First month**	75.54 ± 6.64	66.66 ± 8.56	74.34 ± 12.93	***< 0.01***	*0.83*	***< 0.01***	
**Third month**	77.90 ± 6.30	68.76 ± 7.91	77.82 ± 4.96	***< 0.01***	*0.99*	***< 0.01***	
**P value**	< 0.01*	< 0.01*	< 0.01*				
**APHAB**	**Mean ± SD**			**P value**			
**Pre-HA**	61.54 ± 14.37	62.33 ± 13.46	63.39 ± 12.47	*0.98*	*0.89*	*0.96*	
**First month**	120.18 ± 13.17	99.38 ± 9.41	109.08 ± 13.32	***< 0.01***	***< 0.01***	***0.02***	
**Third Month**	139.00 ± 8.42	123.00 ± 10.92	138.30 ± 9.75	***< 0.01***	*0.96*	***< 0.01***	
**P value**	< 0.01*	< 0.01*	< 0.01*				

n: number of participants, SPIN: speech perception in noise, APHAB: abbreviated profile of hearing aid benefit, * repeated measures anova

### The abbreviated profile of hearing aid benefit (APHAB)

The mean APHAB scores were not statistically significant in the Pre-HA condition for all three fitting groups (F(2, 63) = 0.10, *p* = 0.89), with mean APHAB scores found as 61.54 ± 14.37, 62.33 ± 13.46, and 63.39 ± 12.47 for the NAL-NL2, DSL pediatric, and Hybrid fitting groups, respectively. Significant elevation in APHAB scores were obtained from pre-HA to both the first and third months across all fitting procedures (F(2, 63) = 19.83, *p* < 0.01, *r* = 0.38) Significantly higher APHAB scores were observed at the end of the first month for all fitting groups compared to the pre-HA condition (*p* < 0.01). Specifically, the NAL-NL2 group (120.18 ± 13.17) exhibited significantly higher scores at the first month than both the DSL pediatric (99.38 ± 9.41) and Hybrid fitting (109.08 ± 13.32) groups (*p* < 0.01). The difference between DSL pediatric and Hybrid group was also significant (*p* = 0.02). The third month measurements showed that APHAB scores were significantly lower for the DSL pediatric group (123.00 ± 10.92) compared to both the NAL-NL2 (139.00 ± 8.42) and Hybrid fitting (138.30 ± 9.75) groups (*p* < 0.01). However, there was no significant difference between the NAL-NL2 and Hybrid fitting groups in terms of their third month APHAB scores (*p* = 0.96). Table 2 shows the change of APHAB scores over time and statistical comparison.

## Discussion

This study aimed to evaluate the effects of NAL-NL2, DSL pediatric and the proposed hybrid gain formulas on tinnitus management, SPIN performance, and HA benefit to conclude the best performing formula for the treatment of tinnitus patients with hearing loss. THI and MML results showed that DSL pediatric and hybrid gain fitting procedures were more effective in tinnitus management. However, SPIN performance and hearing benefit were better in the NAL-NL2 and hybrid gain fitting procedures. This study proposed a novel hybrid gain fitting procedure, showing that it provides the best tinnitus management compared to NAL-NL2 and DSL pediatric without compromising SPIN and HA benefit.

In this study, it was shown that the DSL pediatric and hybrid gain formula considering MML and THI scores was the most effective fitting procedure. These fitting procedures significantly reduced THI scores even in the first month post-fitting. A decrease in THI scores of greater than 20 points has been reported to be clinically significant [28]. Here, we observed a reduction of more than 20 points for the three fitting procedures, indicating that HA use is principally effective in tinnitus management, regardless of the fitting procedure. Restoring auditory input with HAs can be expected to regulate auditory plasticity [29]. Previous studies have suggested that tinnitus is associated with an increase in neural activity in the central auditory system [6, 30]. It has been shown that tinnitus suppression can be achieved by reducing this neural activity [31, 32]. Sound enrichment at hearing-impaired frequencies has been shown to reduce hyperactivity and thus tinnitus suppression by regulating neural activity [18, 19, 33]. In fact, previous study in which non-linear frequency compression was applied to tinnitus patients showed no improvement in their tinnitus [34]. The authors suggested that the reason for this is related to the fact that non-linear frequency compression prevents adequate input to the central auditory system. Therefore, this study supports the importance of sound enrichment in the frequency range of hearing loss to provide tinnitus suppression in tinnitus patients. The average hearing thresholds of participants are found to deteriorate at higher frequencies, which is a common configuration in clinic routine. Ching et al. [13] showed that the DSL pediatric fitting procedure provides more high frequency gain than the NAL-NL2 fitting procedure. Therefore, the DSL pediatric procedure may have provided better sound enrichment than the NAL-NL 2 procedure. Thus, it may have been more effective in suppressing tinnitus by reducing tinnitus-related hyperactivity in the central auditory system in line with the results of the previous study conducted by Shetty and Pottackal [15] and Shekhawat et al. [10].

Participants’ HA benefit and SPIN performance were significantly better with the NAL-NL2 and hybrid gain fitting procedures. Due to their higher phoneme content, high frequencies are known to be important in speech intelligibility, particularly in SPIN [22, 23, 35]. Although the DSL pediatric fitting procedure provided more high frequency gain than NAL-NL 2, SPIN performance was poorer in this fitting procedure. The primary reason for this difference may be that, similar to high frequencies, the DSL pediatric procedure has more low frequency gain compared to NAL-NL 2 [13]. Noise and environmental sounds are dominated by low-frequency sounds [22, 23]. The greater low-frequency gain in the DSL pediatric fitting procedure may have amplified the low-frequency noise component in SPIN test, upward spread of masking high-frequency phonemes and thus decreasing SPIN performance. Similar to SPIN, HA benefit of participants may have affected by environmental sounds in daily life due to greater low-frequency amplification in DSL pediatric fitting procedure. Poorer SPIN performance and HA benefit with DSL pediatric may also resulted from loudness recruitment. Loudness recruitment phenomenon is reported to be common among cochlear hearing losses [36]. The greater gain in DSL pediatric procedure may have caused sounds to be perceived as unpleasant. Vaisberg, Beaulac [37] showed that loudness perception is associated with speech intelligibility. Therefore, a possible unpleasant perception with DSL pediatric may have also affected SPIN and HA benefit due to loudness recruitment.

Our findings indicate that the hybrid gain fitting procedure ensures improved tinnitus management, SPIN and hearing benefit. Since the hearing-impaired high frequencies were fitted based on the DSL pediatric procedure, the hybrid gain procedure may have achieved a similar success to DSL pediatric in tinnitus management. Also, low-frequency component of hybrid gain fitting procedure was fitted based on the NAL-NL2 procedure. Thus, relatively less amplification in these frequencies may have reduced the contribution of noise, while higher amplification in higher frequencies may have increased the high-frequency phoneme content. We speculate that these factors may drive both better SPIN performance and HA benefit. Although HA benefit with the hybrid gain procedure was significantly worse than NAL-NL2 in the first month post-fitting, it compensated by the third month post-fitting. This suggests that patients initially struggle with the enriched high-frequency content in the hybrid gain formula and develop a habituation to it over time. This habituation delay may be due to the different loudness techniques used in the NAL-NL2 and DSL pediatric procedures (loudness-normalization and loudness-equalization respectively). Studies suggest that plastic adaptations in the auditory system may occur as early as the two weeks of HA use, while some neural marks may not be present until 3 months of use [38, 39].

The majority of participants have a hearing loss with a sloping configuration. Tinnitus can also be present in flat or rising hearing loss configurations. In our clinical experience, tinnitus can occur frequently in sloping configurations, but the lack of other configurations is an obvious limitation. It is therefore essential to bear in mind that our results may not be generalizable for all tinnitus patients, rather for high frequency hearing loss. Also, there are numerous etiologies of tinnitus in the literature as well. In cases of hearing loss, hearing loss is not always the only cause of tinnitus. Although we tried to exclude organic etiologies, we may have missed many secondary etiologies (such as somatosensory pathologies, metabolic problems, central pathologies) accompanying hearing loss. If we had only included tinnitus patients with a hearing loss etiology, our results may have been more effective. Another limitation of the current study is that we were unable to assess the various subcomponents of tinnitus. Specifically, the influence of the hybrid formula on the different subcomponents, such as emotional response, cognitive effects, and somatosensory influences, was not analyzed. Future research should aim to evaluate these subcomponents to gain a more comprehensive understanding of how the hybrid formula affects tinnitus as a whole. Finally, while we utilized a first-fit approach according to standard rules and REIR measurements, it is important to acknowledge that the fitting process for hearing aids is often iterative. Experienced audiologists typically make several adjustments to the initial settings based on patient feedback and professional assessment. This personalized approach ensures both the audiologist and the patient are satisfied with the fit and performance of the hearing aids. Future studies should consider incorporating this iterative fitting process to better reflect real-world clinical practices.

## Conclusion

Our study revealed that the DSL pediatric fitting was more effective in tinnitus management, while the NAL-NL2 fitting was superior in SPIN and HA benefit. However, improved performance was achieved with the hybrid gain formula without sacrificing tinnitus management, SPIN and HA benefit. Considering that there is no definitive treatment for tinnitus, we incentivize clinicians to use the hybrid gain formula due to its success in tinnitus management, SPIN, and HA benefit.

## Electronic supplementary material

Below is the link to the electronic supplementary material.

Supplementary Material 1

Supplementary Material 2

## Data Availability

The data that support the findings of this study are available from the corresponding author (H. C), upon request.

## Abbreviations

SPIN: Speech Perception in Noise SPIN
NAL-NL: National Acoustical Laboratories’ non-linear
DSL: Desired Sensation Level
APHAB: Abbreviated Profile of Hearing Aid Benefit
THI: Tinnitus Handicap Inventory
HA: Hearing Aid
MML: Minimum Masking Level
REIR: Real Ear Insertion Response
REUR: Real Ear Unaided Response
ISTS: International Speech Test Signal
PTT: Pure Tone Thresholds
