# Supplementary material for: Performance of hybrid gain formula versus traditional fitting formulas in hearing aid fitting in tinnitus patients with hearing loss
Source: Eur Arch Otorhinolaryngol. 2024 Jul 28;281(12):6295–302. doi: 10.1007/s00405-024-08846-z (PMC11564385; doi:10.1007/s00405-024-08846-z)
Supplement: Supplementary file 1 — Supplementary Material 1 [file 405_2024_8846_MOESM1_ESM.docx]

# Table 3. Baseline (Pre-Hearing aid) tinnitus measures and SPIN performances of the participants.

| Fitting Procedure | MML (SPL) Mean ± SD | MML (SPL) P value | THI Mean ± SD | THI P value | SPIN (%) Mean ± SD | SPIN (%) P value | APHAB Mean ± SD | APHAB P value |
| --- | --- | --- | --- | --- | --- | --- | --- | --- |
| NAL-NL2 | 52.27±12.79 | 1.0 | 58.72±13.30 | 0.97 | 60.18±6.23 | 0.89 | 61.54±14.37 | 0.98 |
| DSL Pediatric | 52.14±15.69 | 1.0 | 57.71±13.46 | 0.57 | 59.23±7.54 | 0.96 | 62.33±13.46 | 0.89 |
| Hybrid | 52.17±17.04 | 1.0 | 54.17±18.24 | 0.72 | 60.69±6.89 | 0.76 | 63.39±12.47 | 0.96 |
