# Supplementary material for: Performance of hybrid gain formula versus traditional fitting formulas in hearing aid fitting in tinnitus patients with hearing loss
Source: Eur Arch Otorhinolaryngol. 2024 Jul 28;281(12):6295–302. doi: 10.1007/s00405-024-08846-z (PMC11564385; doi:10.1007/s00405-024-08846-z)
Supplement: Supplementary file 2 — Supplementary Material 2 [file 405_2024_8846_MOESM2_ESM.doc]

**Cover Letter**

17.05.2024

Dear editor,

We are pleased to submit an original research article for consideration for publication in your journal, entitled “Performance of Hybrid Gain Formula Versus Traditional Fitting Formulas in Hearing Aid Fitting in Tinnitus Patients with Hearing Loss”.

We believe that this paper will be of interest to tinnitus researchers and will add value to their clinical practice.

In this paper, we aim to search for the best hearing aid fitting in tinnitus patients with hearing loss. We compared NAL-NL2, DSL pediatric and a novel hybrid fitting procedure (covering both NAL-NL2 and DSL pediatric formulas) in terms of tinnitus management, speech perception in noise (SPIN), and hearing aid benefit. We show that DSL pediatric and hybrid procedures are more successful in tinnitus management than NAL-NL2. However, NAL-NL2 and hybrid procedures have better findings in SPIN and hearing aid benefit. Thus, we concluded that a hybrid fitting procedure provides the best tinnitus management without compromising SPIN and hearing aid benefit. To the best of our knowledge, this is the first study to suggest that hybrid fitting is the optimal fitting procedure for tinnitus patients with hearing loss.

We believe that this manuscript is appropriate for publication in this journal because it demonstrates how hearing-impaired tinnitus patients should have an optimal hearing aid fitting.

We have no conflicts of interest to disclose.

Please address all correspondence concerning this manuscript to me at h.colak2@newcastle.ac.uk

Thank you for your consideration of this manuscript.

Sincerely,

Hasan Çolak

Biosciences institute, Newcastle University,

Newcastle Upon Tyne, United Kingdom
